# The Interaction of CDH20 With β-Catenin Inhibits Cervical Cancer Cell Migration and Invasion via TGF-β/Smad/SNAIL Mediated EMT

**DOI:** 10.3389/fonc.2019.01481

**Published:** 2020-01-09

**Authors:** Chao Li, Hongfeng Ao, Guofang Chen, Fang Wang, Fang Li

**Affiliations:** ^1^Clinical and Translational Research Center, Shanghai First Maternity and Infant Hospital, Tongji University School of Medicine, Shanghai, China; ^2^Department of Pathology, Shanghai Fengxian District Central Hospital, Shanghai Jiao Tong University Affiliated Sixth People's Hospital South Campus, Shanghai, China; ^3^Department of Gynecology, Shanghai East Hospital, Tongji University School of Medicine, Shanghai, China

**Keywords:** cervical cancer, cadherin 20, β-catenin, cancer metastasis, epithelial-to-mesenchymal transition, pSmad2/3

## Abstract

Cancer-associated cadherin 20 (CDH20) is a novel identified cadherin that is genetically altered in several types of human cancer, including cervical cancer. However, its involvement in the progression of cervical cancer remains unknown. In this study, we show that CDH20 was downregulated in clinical cervical cancer samples and its expression correlated with cervical cancer clinical features. CDH20 negatively regulated the migration and invasion of cervical cancer cells. CDH20 increased the expression and promoted the cytoplasm and membrane translocation of β-catenin, and interacted with β-catenin. Mechanistically, CDH20/β-catenin suppressed transforming growth factor-β (TGF-β)-induced epithelial-to-mesenchymal transition (EMT) by downregulating Snail through reducing the phosphorylation and nuclear translocation of Smad2/3. Taken together, our data suggest that CDH20 may act as a tumor suppressor that interacts with β-catenin to inhibit cervical cancer cell migration and invasion via TGF-β/Smad/Snail mediated EMT.

## Introduction

Cervical cancer is one of the most common gynecological malignancies with high morbidity and mortality. A key risk factor underlying cervical cancer is the infiltration of para-uterine tissue, which usually appears in the advanced stages of the disease and strongly affects the quality of life and survival of patients (1). Therefore, a better understanding of the molecular mechanisms involved in cervical cancer metastasis will provide more effective treatment and control for cervical cancer patients.

Tumor metastasis is a multi-stage procedure such as adhesion, migration, and invasion (2). Decreased adhesive contact between tumor cells is critical for local invasion of tumors. Many types of cell adhesion molecules control the cell-to-cell physical interactions. The cadherins (CDHs), a large superfamily of cell-cell adhesion molecules, dynamically regulate adhesive contact to affect tumor invasion and metastasis. Mutation or loss of *cdh* genes has previously been described to occur in many cancers, such as cervical cancer, gastric cancer, and breast cancer (3, 4). For example, E-cadherin, the prototypic member of the CDHs, is renowned for its potent malignancy suppressing activity. Reduction in membranous staining of E-cadherin is found to be significantly correlated with the cervical cancer grade (4). Actually, consistency of the reduction of E-cadherin has even been found in precancerous lesions such as high-grade squamous intraepithelial lesion (SIL) (5). Another important CDH is N-cadherin; malignant cells that shift their expression from E-cadherin to N-cadherin facilitate metastatic dissemination (6).

Dysregulation of cell-cell adhesion components such as E-cadherin/N-cadherin can induce the process of epithelial-to-mesenchymal transition (EMT) (7), which is strongly associated with tumor metastasis (8). Through EMT, the expression levels of epithelial marker genes such as α-catenin and Claudin-3 are decreased, while the expression levels of interstitial marker genes such as vimentin and N-cadherin are increased. In addition, transcription inhibitors of E-cadherin, including Snail (Snail-1), Slug (Snail-2), ZEB1, and Twist, are likely to be affected (9). Of these, Snail is a major transcription inhibitor of EMT that is upregulated in relation to cancer metastasis and recurrence (10). Importantly, the expression of Snail is induced by Smad-mediated phosphorylation in various cancer cells (11).

Cadherin 20 (CDH20) is a type II classical cadherin linked with cell-to-cell adhesion. It has profound effects on neural tube segmentation and neural circuit establishment (12). Previous studies have shown that CDH20 is mutated in several cancers, including esophageal adenocarcinoma (13), colorectal cancer (14), cervical cancer (15), and breast cancer (16). For instance, a copy-number loss of CDH20 is detected in 41% of esophageal adenocarcinoma tissues (13). Moreover, CDH20 has been identified as a high-frequency mutated gene in breast cancer and colorectal cancer (14, 16). However, the exact role of CDH20 in cadherin-mediated adhesion is not certain, and there is no evidence that CDH20 mediates a direct link to cervical cancer metastasis.

In the present study, we evaluated the correlation between aberrant expression of CDH20 and tumor progression in clinical cervical cancer samples. We also examined the effects of CDH20 on cervical cancer cell functions *in vitro*. We hypothesized and validated that CDH20 inhibits the process of EMT and cell migration and invasion via interacting with β-catenin, which suppressed TGF-β-induced EMT by downregulating Snail through reducing the phosphorylation and nuclear translocation of Smad2/3. These observations provide the first evidence that CDH20 may act as a tumor suppressor in human cervical cancer.

## Methods

### Patients and Tissue Samples

Study participants were recruited at the Department of Gynecology, Shanghai First Maternity and Infant Hospital, from April 2013 to December 2013. A total of 48 cervical disease patient specimens were acquired at the time of surgery or biopsy. Patients who were pregnant, had known immunosuppressive diseases or received immunosuppressive therapy, chemotherapy, radiotherapy, or other related antitumor therapies were excluded from the study. All diagnoses were confirmed by at least two certified pathologists following the International Union Against Cancer Guidelines. The overall survival (OS) rate was defined as the period from surgery to death from any cause. Disease-free survival (DFS) rate was defined as the interval from surgery to recurrence/metastasis or death. Clinicopathological characteristics of patients are summarized in Table 1. Samples were frozen and stored within 15 min of removal for quantitative real-time polymerase chain reaction (qRT-PCR) and Western blot analyses.

**Table 1:** The correlation between CDH20 expression and the clinical features (*n* = 48).

**Characteristic**	**Total**	**CDH20 expression**		**χ** ^**2**^	***P*-value**
		**Low (*n* = 37)**	**High (*n* = 11)**		
**Age (years)**				3.51	0.173
≤29	1	0	1		
30–49	36	28	8		
≥50	11	9	2		
**Tumor histology**				1.93	0.381
Squamous	41	33	8		
Adenocarcinoma	5	3	2		
Adeno-squamous	2	1	1		
**Histologic grade**				7.16	0.028^*^
Well	2	0	2		
Moderate	28	22	6		
Poor	18	15	3		
**FIGO stage**				8.49	0.015^*^
I	10	4	6		
II	22	20	2		
III	12	10	2		
IV	4	3	1		
**Tumor size (cm)**					
<4	19	15	4	0.06	0.806
≥4	29	22	7		
**Lymph nodes**				6.68	0.01^*^
Negative	39	33	6		
Positive	9	4	5		

*FIGO, International Federation of Gynecology and Obstetrics. Statistical analyses were carried out using Pearson χ^2^ test*.

* *P < 0.05 was considered significant*.

This study was approved by the Scientific and Ethical Committee of the Shanghai First Maternity and Infant Hospital affiliated with Tongji University. Written informed consent was obtained from each participant after they received detailed explanations of the study objectives and procedures. All experiments were performed in accordance with the ethical standards described in the 1964 Declaration of Helsinki and its later amendments or comparable ethical standards.

### Materials and Chemicals

Recombinant human transforming growth factor-β (TGF-β) was purchased from R&D Systems (Minneapolis, MN, USA). T_4_ DNA ligase and restriction enzymes were obtained from New England Biolabs (Beverly, MA); fibronectin (FN, ≥95.0%) was purchased from Sigma-Aldrich (St. Louis, MO, USA); DAPI was purchased from Beyotime Biotechnology (Jiangsu, China); and protein A/G-agarose beads were purchased from Thermo Scientific (Rockford, IL, USA). Oligonucleotide biosynthesis was done by Generay Biotech Co., Ltd. (Shanghai, China). All the other reagents were of analytical grade and were available from commercial sources.

### Immunohistochemistry (IHC)

Paraffin-embedded cervical tissue samples were obtained from Superbiotek Company. The treatment of tissue sections were carried out as previously described (5). Primary antibodies against CDH20 (1:100 dilution) or β-catenin (1:500 dilution) were used. All sections were slightly counterstained with hematoxylin. The IHC results were graded according to the staining intensity of immunostaining and the percentage of positive tumor cells, as previously described (17). Specifically, the intensity of staining was estimated on a scale from 0 to 3, where 0 = no staining, 1 = weak, 2 = moderate, and 3 = strong, and the extent score ranged from 0 to 3, where 0 = no positive cells, 1 = positive cells < 10%, 2 = 10–50%, 3 = positive cells >50%. The final score was calculated as the multiplication of the two scores of the CDH20 protein levels, with grading from 0 to 9, where (score = 0–1 means negative, score = 2–4 means weak, and score = 6–9 means strong.

### Cell Culture

Human cervical cancer cell lines SiHa (ATCC HTB-35), HeLa (ATCC CCL-2), C-33A (ATCC HTB-31), Ect1/E6E7 (ATCC CRL-2614) and Caski (ATCC CRM-CRL-1550), and the 293T (ATCC ACS-4500) cell line were purchased from the American Type Culture Collection (ATCC) and were authenticated by short tandem repeat (STR) tests. SiHa/C-33A, HeLa/293T, Ect1/E6E7, and Caski cells were cultured in MEM, DMEM, EMEM (MEM plus NEAA), and RPMI 1640 medium (HyClone, Logan, UT) containing 10% of fetal bovine serum (FBS, Gibco, NY, USA) and 1% penicillin/streptomycin (Gibco, NY, USA). Cells were grown in a humidified 5% CO_2_ incubator at 37°C.

### Lentivirus Production and Cell Infection

shRNAs (shRNA#1 and #2) against human CDH20 and a negative control (shCtrl) were synthesized by Shanghai GeneChem Co. Ltd. (Shanghai, China) and then annealed and ligated with pGCSIL-GFP. A human CDH20 knockdown stable cell line was constructed according to a previous study (18). The human CDH20 gene was cloned into pGC-FU to generate pGC-FU-CDH20; the human β-catenin gene was cloned into pGC-FU to generate pGC-FU-CTNNB (GeneChem, Shanghai, China).

Lentiviruses were produced by 293T cells according to the manufacturer's instructions. The empty vector was infected into cells and served as the control. Briefly, 1.5 × 10^6^ 293T cells were seeded onto a 6-cm plate and co-transfected with the recombinant vector (pGC-FU-CDH20 or pGC-FU-CTNNB), and vectors pHelper 1.0 and pHelper 2.0, using Lipofectamine 3000 (Invitrogen, CA, USA). After 16 h, the infection solution was removed, and fresh complete medium was added. The cell supernatants containing lentiviruses were harvested 48–72 h post-transfection. Then, the lentiviruses were used to infect cervical cancer cells with polybrene (8 μg/mL; Sigma-Aldrich). A total of 1 × 10^5^ cells in 2 mL of medium were infected with ~1 mL of lentivirus supernatant. Cells infected with empty vector were used as a control.

### Subcellular Localization of β-Catenin

Nuclear and cytoplasmic extracts from cervical cancer cells were obtained by using NE-PERTM nuclear and cytoplasmic extraction reagents (Pierce) following the manufacturer's instructions. Similarly, membrane and cytosol extracts were obtained using a Membrane and Cytosol Protein Extraction Kit (Beyotime Biotechnology, Jiangsu, China). Twenty micrograms of total protein from each preparation was separated by SDS-PAGE and then immunoblotted with an anti-β-catenin antibody to detect the expression. GAPDH (Santa Cruz Biotechnology, CA, USA), histone H2A (Bioworld Technology, Nanjing, China), and ATP1A1 (Bioworld Technology, Nanjing, China) were used to normalize the total protein expression, the nuclear, and membrane fractions in the lysates, respectively.

### Quantitative Real-Time Polymerase Chain Reaction (qRT-PCR)

Total RNA was extracted from cultured cells or tissues using TRIzol reagent (Invitrogen, Carlsbad, CA) and was then incubated with DNase I. A 500 ng sample of total RNA was used for cDNA synthesis with the Two-Step PrimeScript miRNA cDNA Synthesis Kit (Yeasen, Shanghai, China) and an ABI 7500 Real-Time PCR system (Applied Biosystems, Foster City, CA, USA). qRT-PCR was carried out using a SuperReal PreMix Plus (SYBR Green) Kit (Yeasen, Shanghai, China) with a Light Cycler system. The relative RNA expression level after normalization to GAPDH was calculated in accordance with the change in expression using the equation 2-ΔΔCt. The primers used for qRT-PCR analysis are listed in Table S1.

### Western Blot Analysis

Tissues and total cell lysates were prepared in cell lysis buffer (for Western and IP) (Beyotime Biotechnology, Jiangsu, China) supplemented with a protease inhibitor cocktail (Roche, Basel, Switzerland) and phenylmethylsulfonyl fluoride (Sigma-Aldrich, St. Louis, MO, USA). The protein concentrations were quantified using a BCA protein assay kit (Beyotime Biotechnology, Jiangsu, China) following the manufacturer's instructions. Equal amounts of protein were separated by SDS-PAGE and blotted onto polyvinylidene difluoride (PVDF) membranes (Millipore, Billerica, MA, USA). The blotted membranes were incubated with the indicated doses of primary antibodies against CDH20, β-catenin, E-cadherin, N-cadherin, Vimentin, Snail, Smad2, Smad3, pSmad2, and pSmad3 (Abcam, London, UK) at 4°C overnight, and further incubated with a goat anti-rabbit IgG secondary antibody (Abcam, London, UK) for 1 h. An anti-GAPDH antibody was used as a loading control. Immunoreactive bands were detected with an enhanced chemiluminescence (ECL) Kit (MilliporeSigma, Burlington, MA, USA) and visualized with a FluorChem E imaging instrument (ProteinSimple, San Jose, CA, USA).

### Cell Proliferation Assay

To assess the effects of gene knockdown or overexpression on SiHa or Caski cell proliferation, cells were seeded in a 96-well plate. After attachment, the cells were starved for 8 h and then incubated with complete medium. Cell proliferation was monitored using the Cell Titer 96 AQueous One Solution Cell Proliferation Assay (MTS) (Promega, USA). Briefly, 20 μL MTS was added to each well at 24, 48, or 72 h. Following incubation at 37°C for 3 h, the absorbance at 490 nm was measured with a MRX II microplate reader (Dynex Technologies, Chantilly, VA, USA).

### Transwell Migration and Invasion Assays

Transwell migration and invasion assays were implemented using a chamber containing a polycarbonate filter with a pore size of 8.0 μm (24-well insert; Corning, NY, USA). Polycarbonate filters pre-coated with Matrigel matrix (BD Biosciences, CA, USA) were applied for the invasion assay, along with uncoated filters for the migration assay. Cells were serum-starved for 8 h, and approximately 3–5 × 10^4^ cells were resuspended in 300 μL of serum-free medium and added to the upper chamber. The lower chambers were filled with 800 mL of the indicated medium. After incubation for 16 h at 37°C and 5% CO_2_, the lower chambers were incubated with calcein-AM (Thermo Fisher Scientific) to stain the cells that invaded through the Matrigel matrix or the uncoated membrane. The stained cells were counted in four randomly selected visual fields using a CCD camera mounted to an inverted microscope running MetaMorph image analysis software (Molecular Devices, Sunnyvale, CA).

### Adhesion Assay

Approximately 2–4 × 10^4^ cells were seeded into a 96-well plate and incubated for 6 h at 37°C. After incubation, cells were washed with PBS and fixed with absolute ethanol, followed by staining with crystal violet (0.05%; Fisher Scientific) for 30 min. Then, cells were water-washed and solubilized with 10% acetic acid. Cell adhesion was recorded by reading the absorbance at 570 nm. To estimate fibronectin cell-matrix adhesion, the 96-well plate was pre-coated with 20 μg/mL fibronectin at 4°C for overnight. The wells were blocked with 1% bovine serum albumin (Sigma-Aldrich, St. Louis, MO, USA) for 30 min before seeding cells on it.

### Immunofluorescence Staining

Cell suspensions were placed onto a 35-mm glass bottom plate (Nest, China) at ~40% confluent density. After overnight incubation, the cells were washed three times with ice-cold PBS, fixed with 4% paraformaldehyde for 20 min, and then permeabilized with 0.2% Triton X-100 for another 10 min. Subsequently, the cells were blocked with 5% bovine serum albumin for up to 2 h at room temperature, and incubated with appropriate concentrations of primary antibodies against β-catenin, E-cadherin, N-cadherin, ZO-1, Flag (CDH20) (Abcam, London, UK), Smad3, and pSmad3 (1:100 dilution) overnight at 4°C. Then, the cells were incubated with Alexa Fluor® 488/594 conjugated goat anti-rabbit IgG (Abcam, London, UK, 1:200 dilution) or goat anti-mouse IgG (Abcam, London, UK, 1:200 dilution) secondary antibodies for 1 h. Cell nuclei was stained with DAPI. The stained cells were observed under a fluorescence confocal microscope.

### Immunoprecipitation

Cells were lysed using cell lysis buffer (for Western and IP) plus 1 mM phenylmethanesulfonyl fluoride (PMSF) (Beyotime Biotechnology, Jiangsu, China). Cell lysates were incubated with the primary antibody β-catenin overnight at 4°C. Protein A/G-agarose beads were then added and incubated for 3 h at 4°C. After that, the beads were washed three times with cell lysis buffer and eluted with 5× loading buffer. The proteins were separated by SDS-PAGE and examined by Western blotting.

### Statistical Analysis

The graphical data are presented as the means ± SEM of three independent experiments unless otherwise stated. Statistical analyses were performed using GraphPad Prism 7.0 (GraphPad Software, CA, USA) with one-way ANOVA followed by Bonferroni's *post hoc* test or using SPSS software (standard version 19.0; IBM) by the Pearson's χ^2^ test. A *P* < 0.05 compared with the control was considered statistically significant.

## Results

### CDH20 Expression Was Downregulated in Human Cervical Cancer Tissues

**Figure 1:**
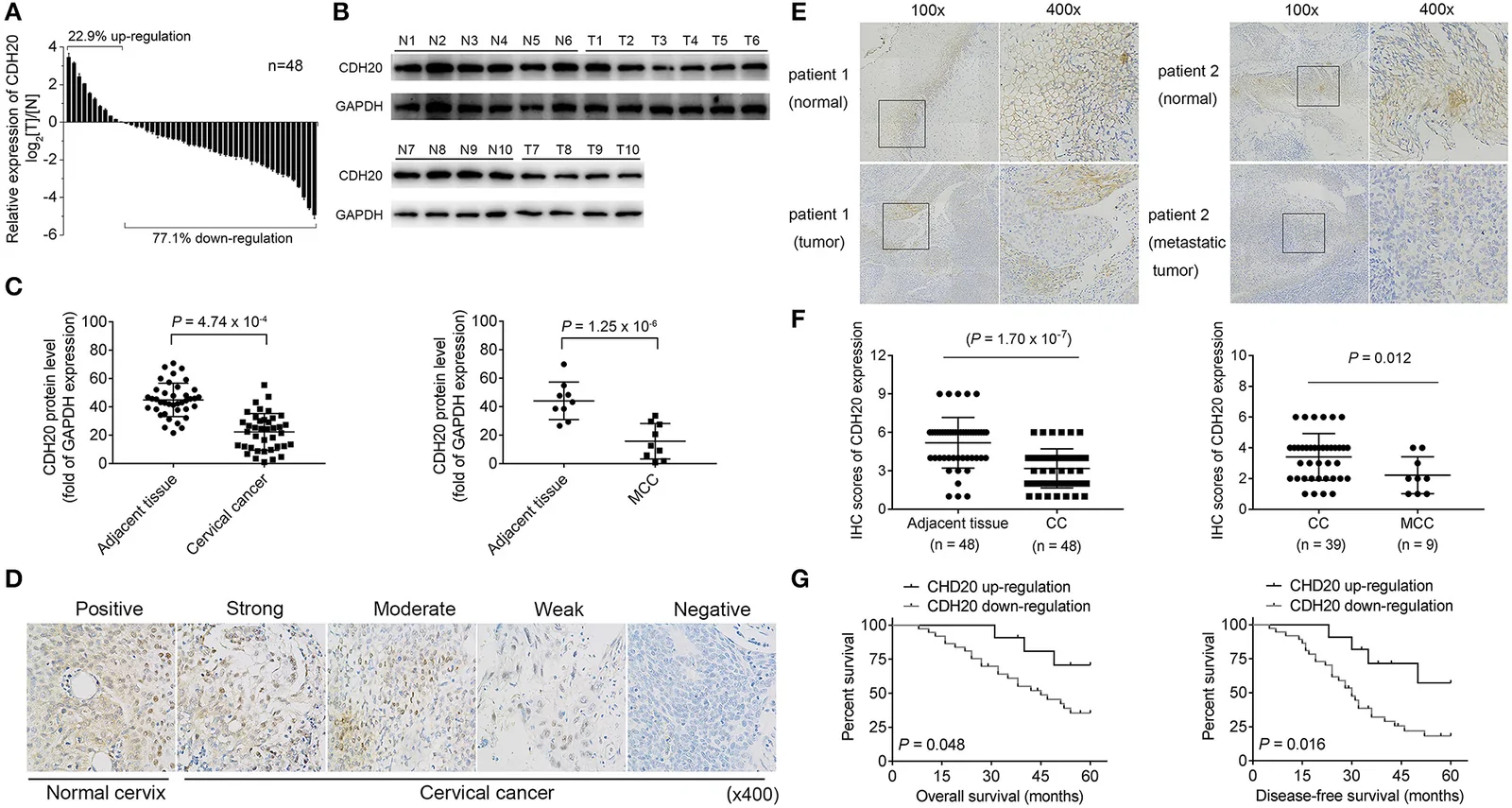
CDH20 expression was downregulated in human cervical cancer samples. **(A)** Levels of CDH20 mRNA in 48 cervical cancer tissues and paired normal adjacent tissues. A Log_2_([T]/[N]) value <0 indicated that CDH20 expression was downregulated in the cervical cancer samples, while a Log_2_([T]/[N]) value >0 indicated that CDH20 expression was upregulated in the cervical cancer samples. Data are presented as the means ± SDs of three independent experiments. **(B)** Representative Western blotting images of CDH20 expression in six paired cervical cancer tissues and four paired cervical cancer with lymphatic metastasis (MCC) tissues. T1–T6, cervical cancer tissues; N1–N6, paired normal adjacent tissues. T7–T10, MCC tissues; N7–N10, paired normal adjacent tissues. **(C)** Statistical analysis of the correlation between CDH20 protein levels in cervical cancer tissues and paired normal tissues (*n* = 39, *P* = 4.74 × 10^−4^) or in MCC tissues and paired normal tissues (*n* = 9, *P* = 1.25 × 10^−6^), as evaluated by Student's *t*-test. Data are presented as the means ± SDs. **(D)** Immunohistochemistry (IHC) staining of CDH20 in normal cervix and cervical cancer tissues. Images, original magnification ×400. **(E)** Representative IHC images of CDH20 expression in cervical cancer tissue and paired normal tissue (patient 1) and in MCC tissue and paired normal tissue (patient 2). Left panel, original magnification ×100; right panel, original magnification ×400. **(F)** The score of CDH20 staining between cervical cancer tissue and adjacent normal tissue, or between cervical cancer tissues with lymphatic metastasis (MCC) and without metastasis. **(G)** Correlation between CDH20 expression and the survival [overall survival (OS) and disease free survival (DFS)] rates of the patients with cervical cancer. Survival curves were constructed using the Kaplan-Meier method and analyzed by the log-rank test. *P* < 0.05 was considered significant.

Previous high-throughput sequencing results indicated that CDH20 is mutated in cervical cancer tissues and has a potential role in cervical disease progression (15). To explore the exact role of CDH20, we first analyzed the level of CDH20 mRNA in 48 paired cervical cancer and matching non-cancerous adjacent tissue samples. As shown in Figure 1A, a reduced level of CDH20 mRNA was observed in 37 (~77.1%) cervical cancer tissues. Moreover, the level of CDH20 protein was negatively correlated with cervical cancer in both nonmetastatic or lymphatic metastatic tumor samples (Figures 1B,C), suggesting that CDH20 was downregulated in cervical cancer.

Immunohistochemical (IHC) analysis of the paired 48 cases revealed that CDH20 was expressed mainly in the nonmalignant tissues, and the staining was significantly stronger than that in the cervical cancer tissues (Figures 1D–F). Compared with the non-metastatic tumor tissues, lower levels of CDH20 protein were observed in the tumors with lymphatic metastasis (Figure 1F, right panel). Moreover, a lower level of CDH20 protein was associated with poor OS and DFS rates (Figure 1G). Furthermore, lower CDH20 protein levels were observed to negatively correlate with histologic grade, FIGO stage, and lymph node metastasis (Table 1). Altogether, these data suggest that CDH20 was downregulated in cervical cancer and might promote the progression of cervical cancer by enhancing the motility of cancer cells.

### CDH20 Downregulation Promotes the Migration and Invasion of Cervical Cancer Cells

**Figure 2:**
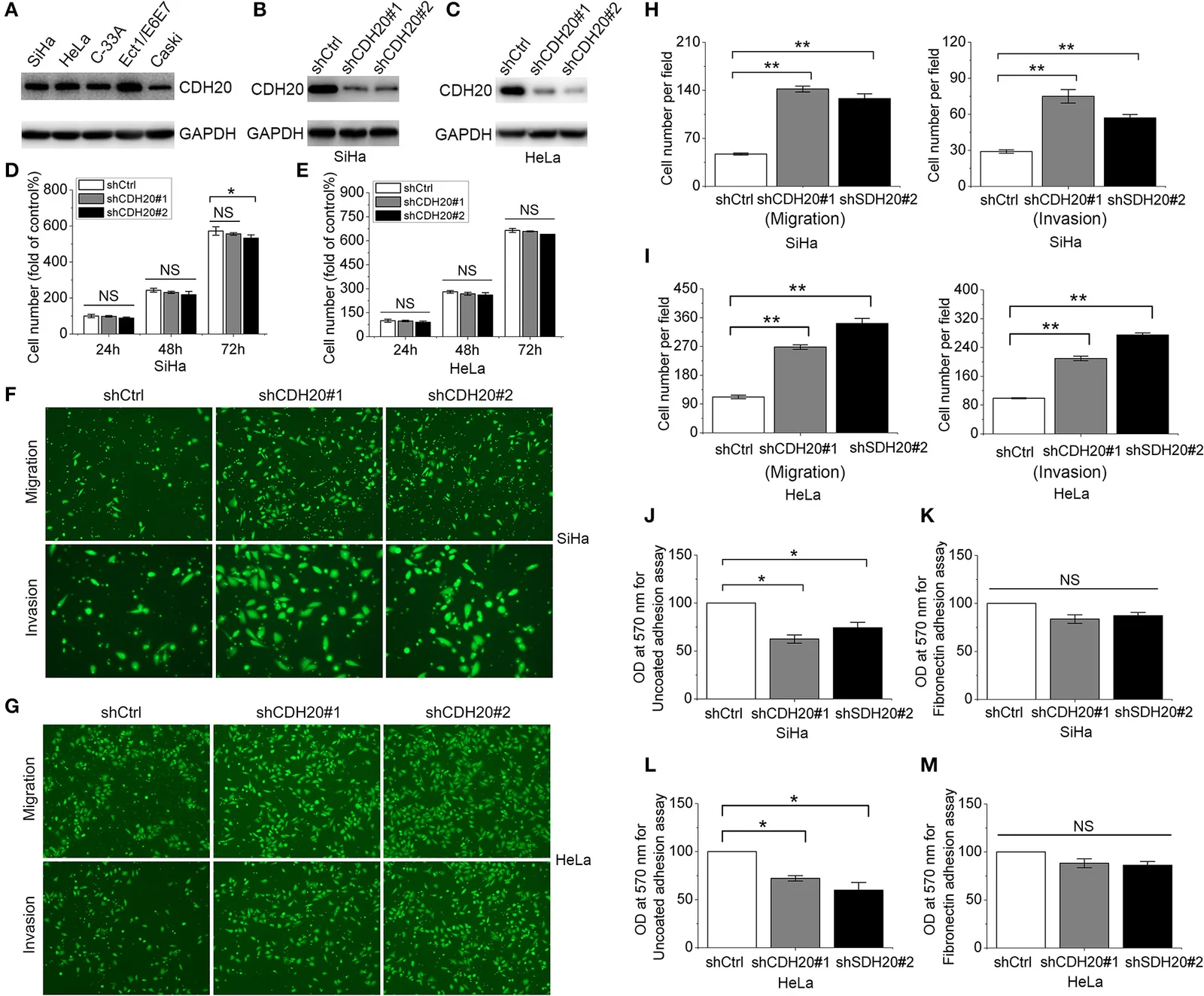
CDH20 plays an important role in cervical cancer cell migration, invasion and adhesion. **(A)** Western blot analysis of CDH20 protein expression in four different cervical cancer cell lines (SiHa, HeLa, C33A, and CaSki) and 1 cervical squamous epithelial cell line (Ect1/E6E7). **(B,C)** Stable SiHa and HeLa cells were established by lentivirus infection with scrambled shRNA (shCtrl) and two specific shRNAs against CDH20 (shCDH20#1 and shCDH20#2). Cells lysates were subjected to Western blot analysis with the indicated antibodies. **(D,E)** Proliferation of the SiHa and HeLa stable cell lines was detected by MTS assay. The cell proliferation index of the shCtrl group at 24 h was defined as 100%. **(F,G)** Migration and invasion of SiHa and HeLa stable cell lines were determined by transwell assay. Representative images are shown. **(H,I)** Changes in cell migration and invasion were quantified. **(J,K)** Cell adhesion to uncoated plates was measured by cell adhesion assay using the SiHa and HeLa stable cell lines. **(L,M)** Cell adhesion to fibronectin-coated plates was measured by cell adhesion assay using the SiHa and HeLa stable cell lines. All statistical data were analyzed by one-way analysis of variance (ANOVA). All values represent the means ± SEM. ^*^P < 0.05, and ^**^P < 0.01 compared with the control. NS, non-significant.

Based on the above results, we speculated that loss of CDH20 function is critical for cervical cancer cell metastasis. To test this assumption, we investigated the role of CDH20 in cervical cancer cell lines. First, we detected CDH20 expression in five different cervical cell lines and found that the selected cervical cancer cell lines had lower expression of CDH20 than the cervical epithelial cells (Ect1/E6E7 cells) (Figure 2A). In addition, CDH20 was highly expressed in SiHa and HeLa cells but was expressed at low levels in Caski cells. Therefore, we chose the SiHa and HeLa cell lines and used two specific shRNAs to reduce CDH20 expression. The knockdown effects of shRNAs on CDH20 protein expression were verified by Western blotting (Figures 2B,C). Then, we performed an MTS assay to detect cervical cell proliferation. Representative results showed a slight decrease in cell number in only one CDH20 knockdown group after 72 h, suggesting that CDH20 downregulation has little effect on cervical cell proliferation (Figures 2D,E). Subsequently, the migration and invasion of cervical cells were assessed by transwell assays. It was observed that decreased CDH20 led to an obvious promotion of cell migration and invasion of 2- to 3-fold (Figures 2F–I). In addition, we determined the effect of CDH20 knockdown on SiHa and HeLa cells adhesion. Cell adhesion was first assessed on uncoated tissue culture plates, and a marked reduction in adhesion was observed (Figures 2J,L). Because fibronectin was reported to strengthen cell adhesion (19), we next examined cell adhesion using a fibronectin matrix. As shown in Figures 2K,M, CDH20 knockdown-induced reduction of cell adhesion was reversed to some extent by fibronectin coating. Taken together, these results suggest that CDH20 inhibits migration and invasion by promoting the adhesion of cervical cancer cells.

### Overexpression of CDH20 Suppresses the Migration and Invasion of Cervical Cancer Cells

**Figure 3:**
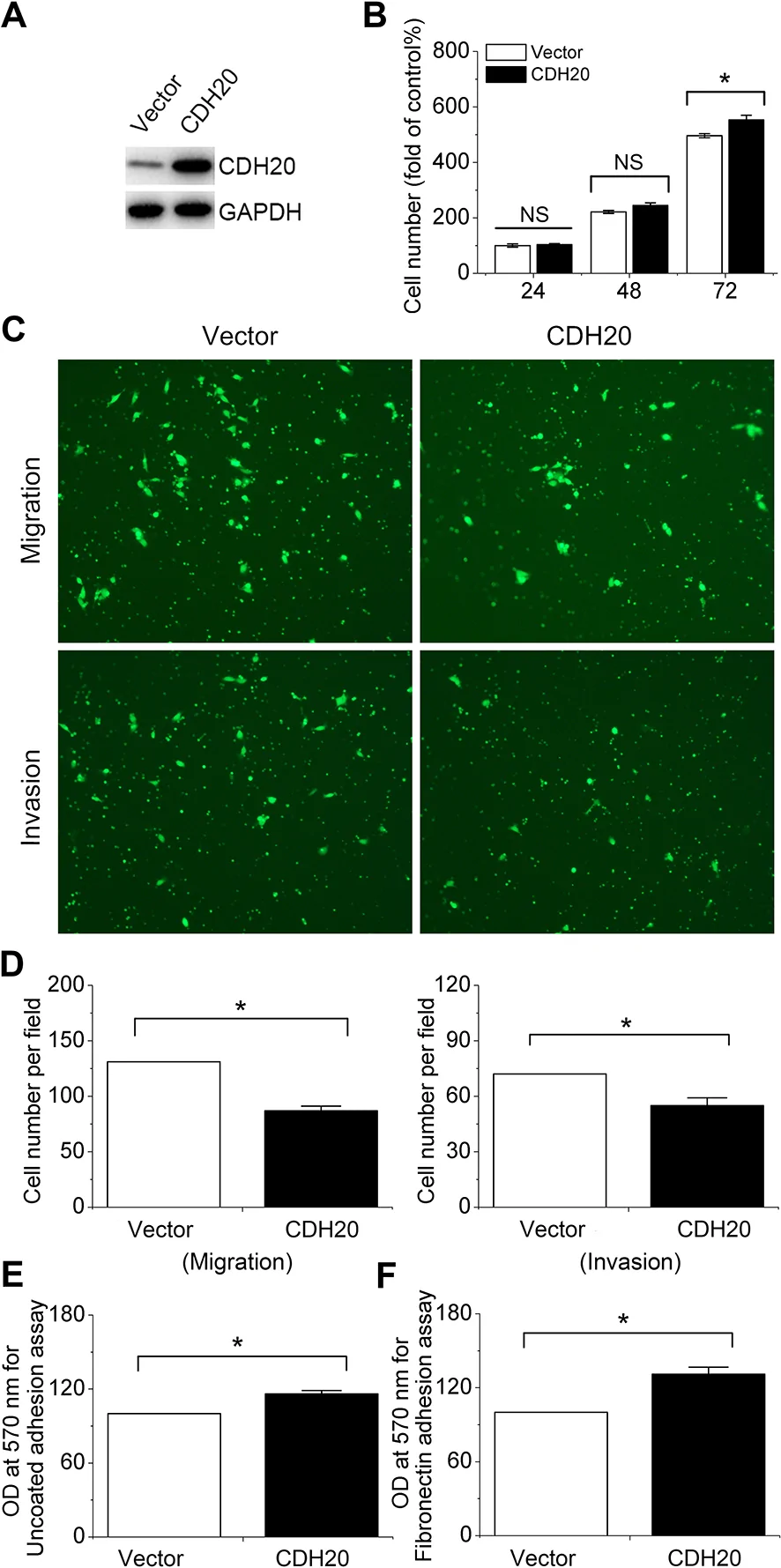
Upregulated CDH20 enhances cervical cell adhesion but reduces cell migration and invasion. Caski cell lines stably overexpressing CDH20 or empty vector were established by lentivirus infection. **(A)** The lysates were blotted with the indicated antibodies. **(B)** Proliferation of the Caski stable cell lines was detected by MTS assay. The cell proliferation index of the vector group at 24 h was defined as 100%. **(C)** Migration and invasion of Caski stable cell lines were determined by transwell assay. Representative images are shown. **(D)** Changes in cell migration and invasion were quantified. **(E)** Cell adhesion to uncoated plates was measured by cell adhesion assay using the Caski stable cell lines. **(F)** Cell adhesion to fibronectin-coated plates was measured by cell adhesion assay using the Caski stable cell lines. All statistical data were analyzed by one-way analysis of variance (ANOVA). All values represent the means ± SEM. ^*^P < 0.05 compared with the control. NS, non-significant.

To verify the important role of CDH20 in cervical cancer cells using another method, we stably overexpressed CDH20 in the Caski cell line (Figure 3A). As shown in Figure 3B, CDH20 upregulation did not affect cervical cell proliferation within the first 48 h; however, the migration and invasion of Caski cells were significantly reduced (Figures 3C,D). Then, we used cell adhesion experiments with uncoated plates or a fibronectin matrix. The overexpression of CDH20 obviously increased cell adhesion, and fibronectin coating further enhanced this effect (Figures 3E,F). These observed results were compatible with those found in CDH20 knockdown cell lines, further supporting the inhibition effect of CDH20 on cell migration and invasion in cervical cells.

### Different Expression Patterns of CDH20 Differently Affect the Epithelial-To-Mesenchymal Transition (EMT) Process of Cervical Cancer Cells

**Figure 4:**
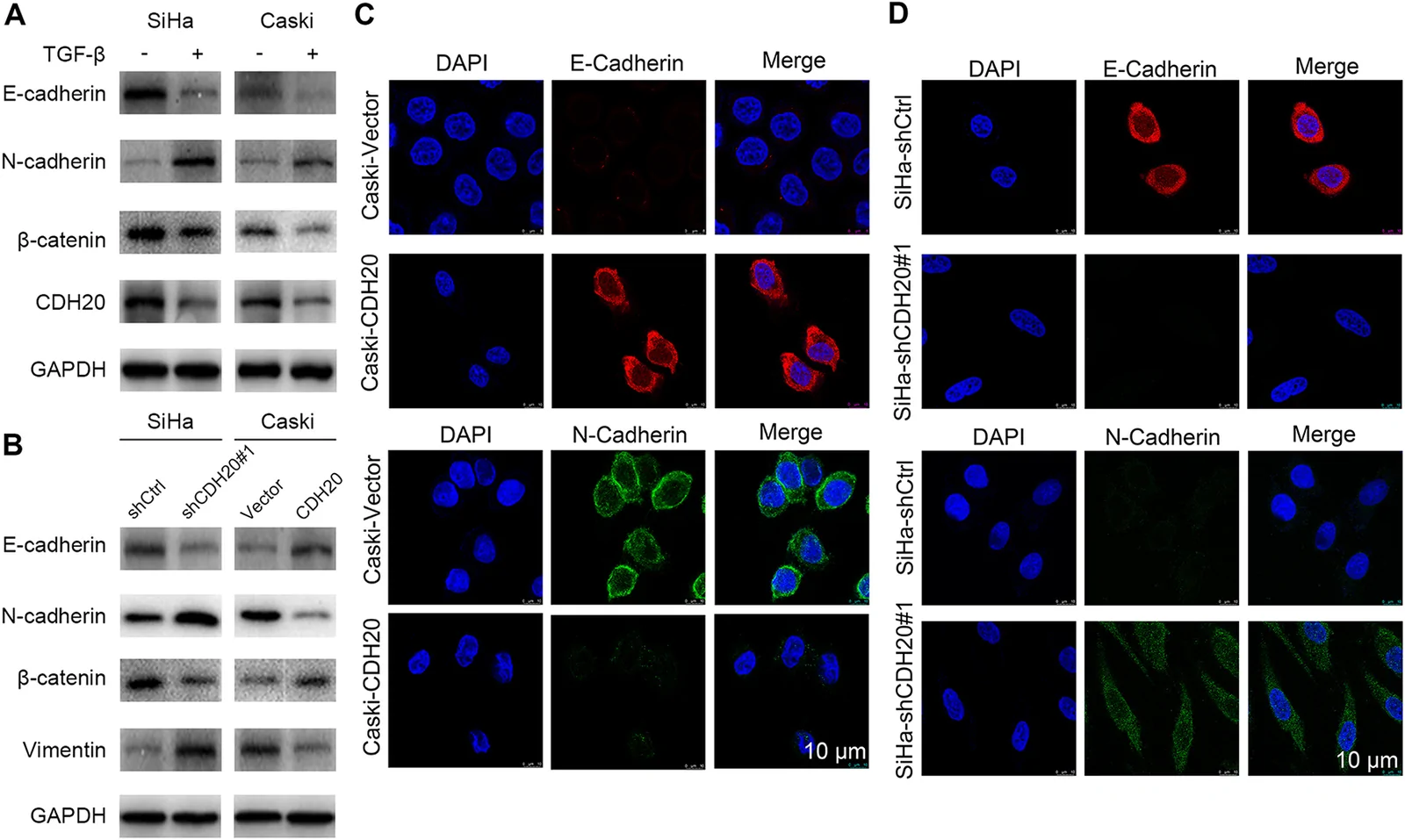
Different expression patterns of CDH20 differently affect the EMT process of cervical cancer cells. **(A)** CDH20 was downregulated in artificial EMT models induced by TGF-β in both SiHa and Caski cells. Upregulation of N-cadherin or downregulation of E-cadherin and β-catenin expression was verified in EMT models. **(B)** Western blot analysis of EMT markers (E-cadherin, N-cadherin, β-catenin, and vimentin) in the CDH20 knockdown SiHa cells or the CDH20 overexpression Caski cells compared with those in the control groups. GAPDH was used to normalize protein expression. One representative of three different experiments, for each of the analyses performed, is shown. **(C)** Immunofluorescence showed that E-cadherin and N-cadherin expression levels were increased or decreased, respectively, in CDH20-overexpressing Caski cells via laser confocal microscopy. **(D)** Immunofluorescence showed that E-cadherin and N-cadherin expression levels were decreased or increased, respectively, in CDH20-knockdown SiHa cells via laser confocal microscopy. Representative images of E-cadherin and β-catenin N-cadherin localization are shown.

TGF-β induced EMT model in human epithelial cells is a commonly used model to dissect the molecular mechanisms of cancer metastasis (20). To examine this effect in cervical cancer, TGF-β was added to SiHa and Caski cell cultures. As shown in Figure 4A, TGF-β notably induced EMT by decreasing E-cadherin and β-catenin and increasing N-cadherin expression in both SiHa and Caski cell lines. Interestingly, the expression of CDH20 was simultaneously reduced. In light of these observations, we hypothesized that CDH20 inhibited EMT, and further examined whether altering the expression of CDH20 could affect the phenotypes of cervical cancer cell lines. The overexpression of CDH20 augmented the levels of E-cadherin and β-catenin and reduced the levels of N-cadherin and vimentin in Caski cells, implying that CDH20 can inhibit the EMT process (Figure 4B). Subsequent confocal immunofluorescence analysis also confirmed the hypothesis (Figure 4C). Moreover, western blot and confocal immunofluorescence analysis suggested that CDH20 knockdown in SiHa cells generated the opposite results (Figures 4B,D).

### CDH20 Promotes the Membrane Accumulation of β-Catenin

**Figure 5:**
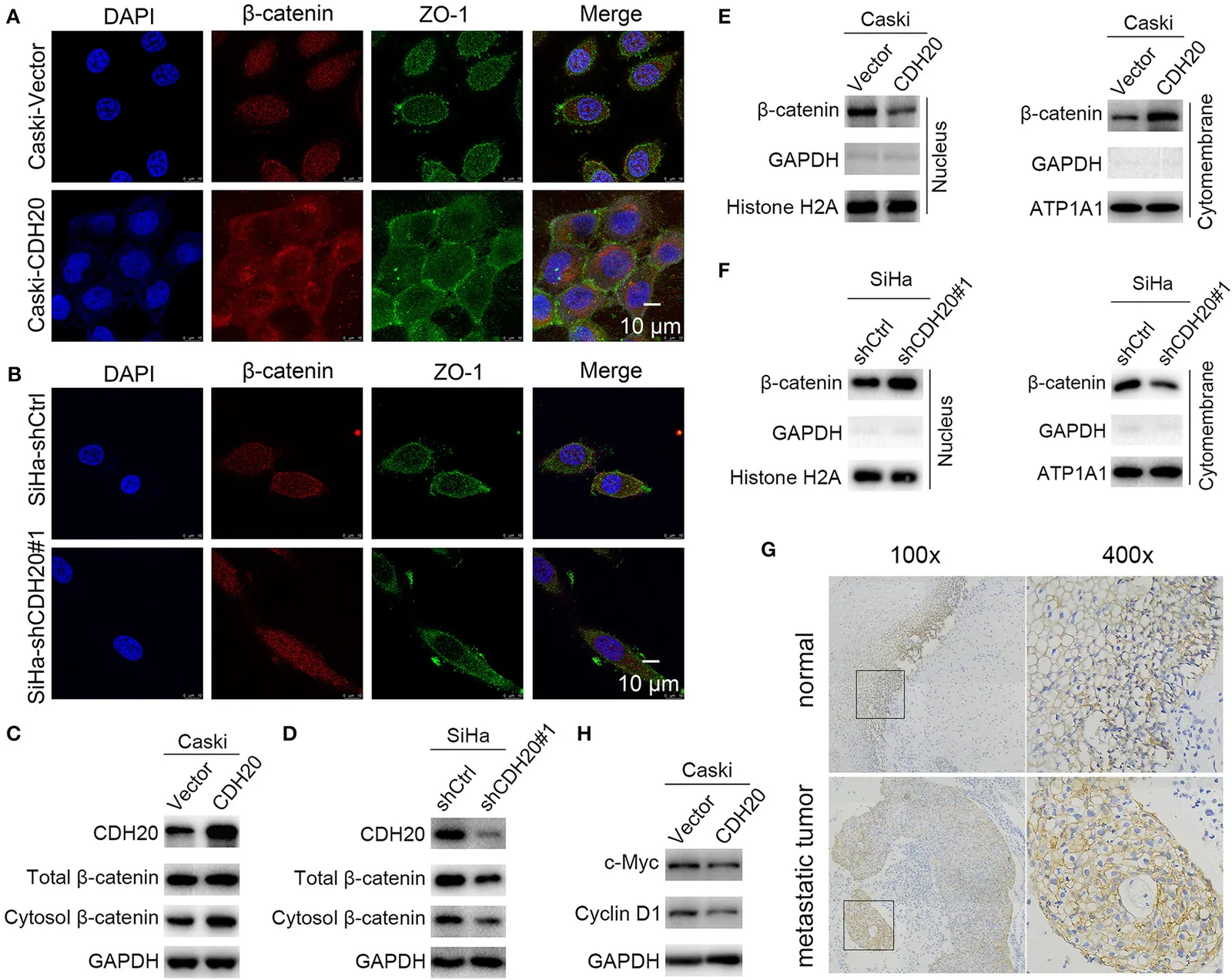
CDH20 promotes the membrane accumulation of β-catenin. **(A,B)** The cellular location of β-catenin was detected by immunofluorescence in CDH20-overexpressing Caski **(A)** or CDH20-knockdown SiHa **(B)** cells. Representative images of β-catenin and ZO-1 localization are shown. **(C–F)** The cellular location of β-catenin was detected by Western blotting. GAPDH, histone H2A, and ATP1A1 were used to normalize the protein expression in total lysates and in the nuclear and membrane fractions, respectively. Caski cells with exogenous CDH20 overexpression **(C,E)** or SiHa cells with CDH20-knockdown **(D,F)** were used. **(G)** Immunohistochemical staining of β-catenin in metastatic cervical cancer and adjacent normal tissues. Left panel, original magnification ×100; right panel, original magnification ×400. **(H)** Western blot analysis of the Wnt/β-catenin pathway-targeted proteins c-Myc and cyclin D1. Caski cells with exogenous CDH20 overexpression or empty vector were used. One representative of three different experiments, for each of the analyses performed, is shown.

Cadherins typically form a complex with β-catenin at the plasma membrane as part of the adherens junctions, while dissociated β-catenin is targeted for ubiquitination and subsequent degradation by the β-catenin degradation complex (21). Intriguingly, β-catenin was observed to partially accumulate on the membrane after overexpression of CDH20 (Figure 5A), while CDH20 knockdown reversed the effect (Figure 5B). This observation prompted us to speculate whether CDH20 affects β-catenin membrane expression in cervical cancer cells. To test this hypothesis, we examined the protein expression of β-catenin in CDH20-overexpressing Caski cells. As shown in Figures 5C,E, CDH20-upregulated Caski cells obviously increased both the total cellular and cytoplasmic or cytomembrane β-catenin but decreased nuclear β-catenin, compared with control cells. In contrast, knockdown of CDH20 was able to generated the opposite results in SiHa cells (Figures 5D,F). Furthermore, the staining distribution of β-catenin in cervical cancer tissues with metastasis was partially transferred to the membrane compared with that in adjacent normal tissues (Figure 5G). Therefore, the results suggest that CDH20 affected both expression and membrane distribution of β-catenin in cervical cancer cells.

As is known, β-catenin is an indicator of Wnt/β-catenin pathway activation. Herein, the increased nuclear β-catenin in the control Caski cells (Figure 5E) suggested that CDH20 might act as a negative regulator of the canonical Wnt signaling. To test this possibility, the expression levels of Wnt/β-catenin target genes were examined. As expected, the protein levels of c-Myc and cyclin D1 were observably reduced upon overexpression of CDH20 (Figure 5H), implying that CDH20 might antagonize the canonical Wnt pathway.

### CDH20 Interacts With β-Catenin to Modulate Cervical Cancer Cell Migration and Invasion

**Figure 6:**
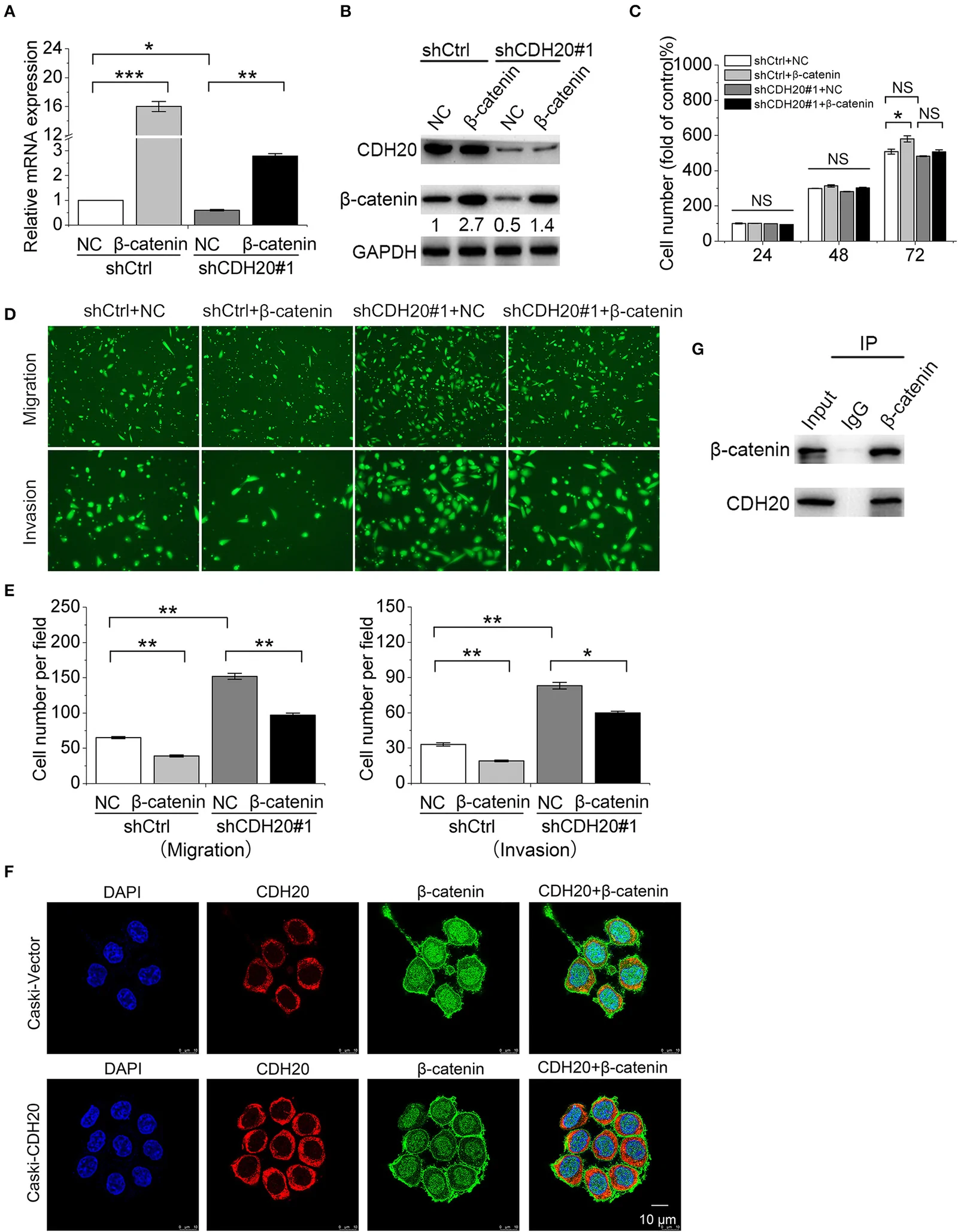
CDH20 interacts with β-catenin to modulate cervical cancer cell migration and invasion. A SiHa stable cell line with/without CDH20 knockdown was transfected with a β-catenin overexpression vector or empty vector for 48 h. **(A,B)** Quantitative real-time polymerase chain reaction (qRT-PCR) **(A)** and Western blot **(B)** analyses of β-catenin/CDH20 mRNA and protein levels in the stable cell lines. Data are presented as the means ± SDs of three independent experiments. **(C)** Proliferation of the SiHa stable cell lines was detected by MTS assay. The cell proliferation index of the control group (shCtrl + NC) at 24 h was defined as 100%. **(D)** Migration and invasion of the stable cell lines were determined by transwell assay. Representative images are shown. **(E)** Changes in cell migration and invasion were quantified. **(F)** Immunofluorescence with CDH20 and β-catenin primary antibodies examining the relative locations of the two proteins in Caski cells. Representative images of CDH20 and β-catenin localization are shown. Scale bar, 10 μm. **(G)** An immunoprecipitation assay was used to examine the interaction between exogenously expressed CDH20 and β-catenin in HEK293T cells. All statistical data were analyzed by one-way analysis of variance (ANOVA). All values represent the means ± SEM. ^*^P < 0.05, ^**^P < 0.01, and ^***^P < 0.001 compared with the control. NS, non-significant. GAPDH was used to normalize protein expression. One representative of three different experiments, for each of the analyses performed, is shown.

To further confirm the importance of CDH20 in the presence of β-catenin, we performed rescue experiments in cervical cancer cells. The β-catenin overexpression plasmid was transfected into CDH20-downregulated SiHa cells, and cell proliferation, migration, and invasion assays were performed. The overexpression effects on β-catenin transcription and protein expression were confirmed by qRT-PCR and Western blotting (Figures 6A,B). The results of the cell proliferation assay showed that increased β-catenin did not have an evident effect on the proliferation of SiHa cells (Figure 6C). In contrast, β-catenin overexpression partially counteracted the promotion effects of CDH20 knockdown on cervical cell migration and invasion (Figures 6D,E).

We next investigated whether the CDH20-mediated synergistic effect with β-catenin involves their physical interaction. First, confocal immunofluorescence analysis was performed. CDH20 was mainly located on the cytoplasm and membrane, while β-catenin was mainly located on the nucleus and membrane. Overexpression of CDH20 enhanced the intensity of these two proteins on the membrane (Figure 6F). To further confirm the interaction between CDH20 and β-catenin in a mammalian system, we co-expressed vectors containing CDH20 and β-catenin in HEK293T cells. The co-immunoprecipitation assays revealed that CDH20 bound to β-catenin (Figure 6G). Thus, the results indicated that CDH20 interacts with β-catenin to inhibit cervical cancer cell migration and invasion.

### CDH20/β-Catenin Suppresses Cervical Cancer Cell EMT and Cell Migration and Invasion Through the TGF-β/Smad/Snail Signaling Pathway

**Figure 7:**
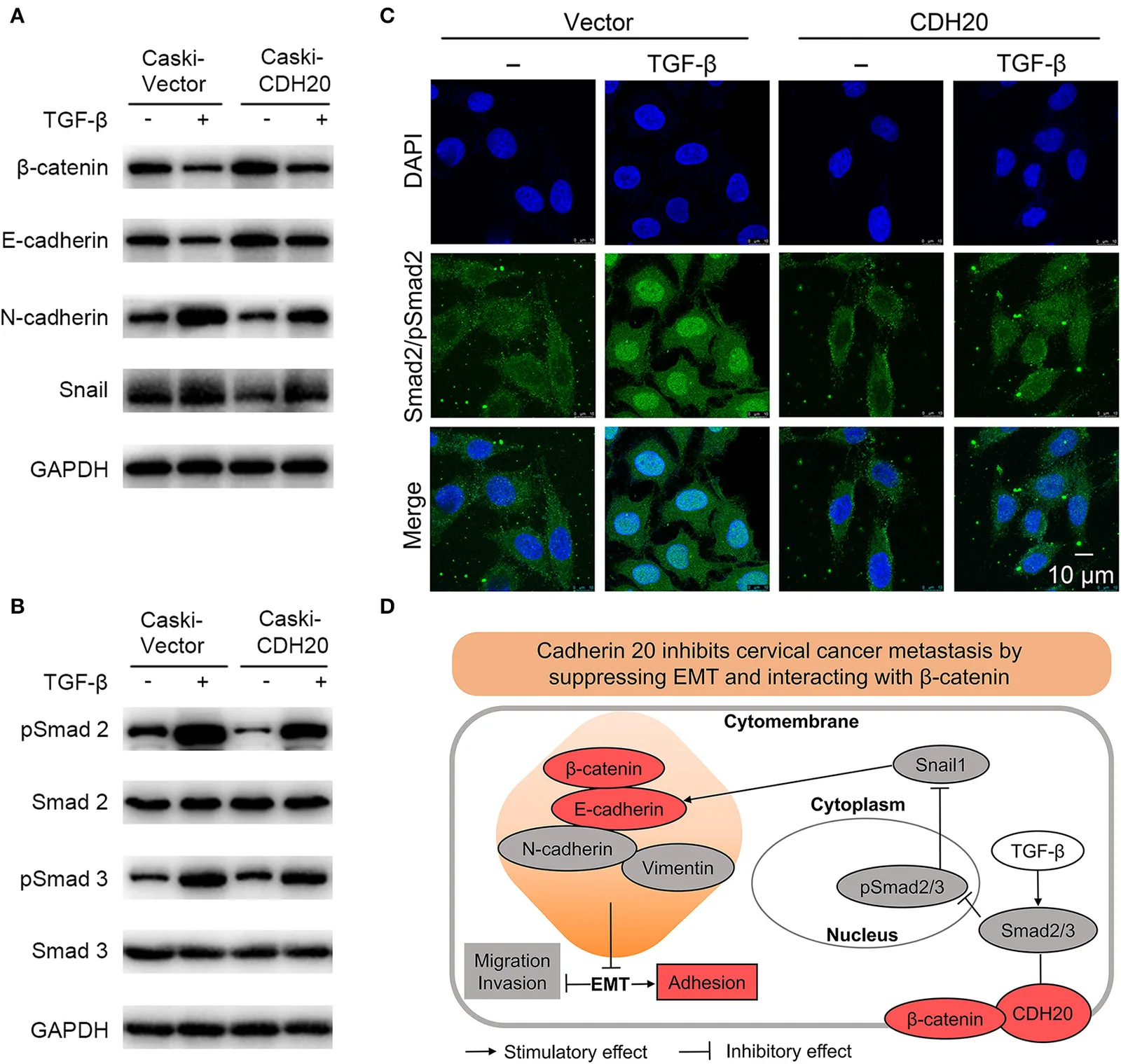
CDH20/β-catenin suppresses cervical cancer cell EMT and metastasis through the TGF-β/Smad/Snail signaling pathway. **(A)** Western blot analysis suggested that TGF-β-induced EMT was weakened by the overexpression of CDH20. **(B)** Western blot analysis showed that TGF-β increased Smad2/3 phosphorylation, while this effect could be attenuated via CDH20 overexpression. GAPDH was used to normalize protein expression. One representative of three different experiments, for each of the analyses performed, is shown. **(C)** Immunofluorescence analysis indicated that overexpression of CDH20 decreased the phosphorylation of Smad2 and interfered with the nuclear translocation of pSmad2. Representative images of Smad2/pSmad2 localization are shown. Scale bar, 10 μm. **(D)** Summary schematic depicting the CDH20-mediated molecular mechanisms in cervical cancer cells. CDH20 directly interacting with β-catenin to regulate the invasion and EMT processes via the TGF-β/Smad2/3/Snail signaling pathway.

To address how CDH20/β-catenin suppresses EMT and migration and invasion in cervical cancer cells, we investigated the causal relationship among CDH20, TGF-β, and EMT. As shown in Figure 7A, increased N-cadherin or Snail and decreased E-cadherin or β-catenin expression were observed in both CDH20-overexpressing and vector control Caski cells in response to TGF-β, suggesting that TGF-β distinctly induced EMT. Considering that Snail expression is induced by Smad 2/3-mediated phosphorylation in various cancer cells (11), we then examined the expression level of Smad 2/3. As shown, Smad2/3 maintained a consistent expression level, and pSmad2/3 was observed with the addition of TGF-β (Figure 7B). Notably, overexpression of CDH20 reduced the level of Snail (Figure 7A), which in turn might have suppressed TGF-β induced EMT by upregulating the expression of E-cadherin. Moreover, CDH20 upregulation reduced the phosphorylation of Smad2/3 to some degree and inhibited the nuclear translocation of pSmad2 (Figures 7B,C). Therefore, these findings indicate that CDH20/β-catenin suppresses EMT via TGF-β/Smad2/3/Snail signaling in cervical cancer cells.

## Discussion

Much effort has been expended toward understanding the molecular basis of tumor progression. Cadherin, a calcium-dependent cell adhesion molecule, has been identified as a key factor in mediating the progression of carcinoma toward the invasive state (22). Metastatic potential is often associated with downregulation of cadherin expression or function (23). Although CDH20 mutations have been found in cervical cancer, the effect of aberrant CDH20 expression on the metastasis of cervical cancer is yet unclear. In this study, we found decreased CDH20 levels in cervical cancer tissues and uncovered that CDH20 is important for cervical cancer cell migration, invasion, and adhesion. Mechanistically, CDH20 interacted with β-catenin to suppress TGF-β-induced EMT by downregulating Snail through reducing the phosphorylation and nuclear translocation of Smad2/3. Therefore, our study implies that dysregulation of CDH20 may functionally drive the metastasis of cervical cancer.

Our results suggest that CDH20 functions as a tumor suppressor in cervical cancer. CDH20 is involved in cell adhesion and has been reported to be genetically altered in several cancers (13–16). Moreover, analysis of osteosarcoma in Paget's disease reveals that the tumor undergoes loss of heterozygosity in the CDH20 region, which is similar to that of the tumor suppressor E-cadherin (24). This is important because the cell adhesion pathway is known to be critical for cancer cell invasion and metastasis, which are the symptoms of advanced cancer. Consistent with these results, we found that CDH20 negatively regulates the migration and invasion of cervical cancer cells and is downregulated in localized or metastatic cervical tumors. Hence, our findings provide direct evidence supporting the hypothesis that CDH20 might be a candidate tumor suppressor gene. These data also stimulate our interest in investigating the roles of CDH20 in other cancers in future work.

The enhanced cell adhesion by CHD20 might result from its increase effect on the membrane expression of β-catenin and direct binding to β-catenin. β-Catenin is a soluble protein that binds to not only E-cadherin but also to the adenomatous polyposis coli (APC) protein, Axin, glycogen synthase kinase-3β (GSK-3β), and T-cell factor/lymphoid enhancer factor (TCF/LEF) (25). In general, cadherins and members of the TCF/LEF family competitively bind to the same domain of β-catenin, thereby, increasing the expression of cadherin could reduce the aggregation of β-catenin in the nucleus and enhance intercellular adhesion. In the present study, we found that the increased β-catenin was located on the cell membrane and not in the nucleus. Further immunoprecipitation confirmed the interaction of β-catenin with CDH20 on the membrane. Therefore, we can speculate that with the increased intracellular levels of CDH20, the process of EMT is reversed, and the expression of E-cadherin and β-catenin is enhanced. The increased β-catenin on the membrane may form complexes with the CDH20 and E-cadherin proteins, thus mediating and strengthening cell adhesion.

EMT inhibition by CDH20/β-catenin through the inactivation of TGF-β/Smad2/3/Snail signaling is an important finding of this study. Cancer cells that undergo EMT may program to invade to facilitate cancer progression and metastasis (26). Nevertheless, the induction of EMT is closely related to the alteration of cadherin (27, 28). In line with prior findings, our data showed that CDH20 inhibited cervical cancer cell migration and invasion, and dysregulation of CDH20 caused changes in EMT-related proteins, indicating that CDH20 was associated with EMT in cervical cancer cells. EMT is triggered by a number of signaling pathways, such as the TGF-β/Smad (29), Notch (30), RTK (31), and Wnt/β-catenin signaling pathways (32). Here, we found that Snail participated in TGF-β-induced EMT, where Snail is confirmed to be a direct target of TGF-β/Smad signaling (33). Consistently, this correlation is further demonstrated in our study because the TGF-β/Smad2/3 pathway was closely related to EMT activation and co-regulated by CDH20. We further revealed that overexpression of CDH20 can reduce the phosphorylation level of Smad2/3 and suppress the nuclear translocation of pSmad2 presumably to regulate Snail transcription. These results suggest that CDH20/β-catenin modulates EMT through the TGF-β/Smad2/3/Snail signaling pathway. The phenomenon that CDH20 overexpression-induced reversal of cell migration and invasion was similar to that induced by E-cadherin overexpression (34).

Although Wnt/β-catenin signaling plays significant roles in the regulation of both EMT and invasive cancer formation (35), it appears to have no direct association with CDH20-mediated functions in our study. β-catenin is competitively utilized by cadherins and the Wnt/β-catenin pathway, in which the expression levels of cadherin and β-catenin are negatively interrelated (36). For instance, increased expression of cadherin lead to antagonize β-catenin signaling activity during mid and late pregnancy, indicating that cadherin may function as an inhibitor of the Wnt/β-catenin pathway (37). Herein, the interaction of CDH20 with β-catenin and the decreased levels of c-Myc and cyclin D1 upon CDH20 overexpression in Caski cells, suggesting that CDH20 negatively regulates the Wnt/β-catenin pathway.

In summary, this study provides the first evidence that CDH20 plays important roles in cervical cancer cell migration, invasion, and adhesion. CDH20 is found to increase the expression of β-catenin and interact with β-catenin on the cytomembrane. The CDH20/β-catenin complex can modulate the expression of EMT-related proteins via the TGF-β/Smad2/3/Snail signaling pathway (Figure 7D). Furthermore, the evaluation of clinical samples shows that dysregulation of CDH20 is associated with both metastatic and non-metastatic cervical cancer. Thus, CDH20 might serve as a potential marker and target for the clinical diagnosis and therapy of cervical cancer.

## Data Availability Statement

All datasets generated for this study are included in the article/Supplementary Material.

## Ethics Statement

The studies involving human participants were reviewed and approved by Scientific and Ethical Committee of the Shanghai First Maternity and Infant Hospital affiliated with Tongji University. The patients/participants provided their written informed consent to participate in this study.

## Author Contributions

CL and FL designed research and revised the paper. CL and HA analyzed data and wrote the paper. CL, HA, GC, and FW performed research.

### Conflict of Interest

The authors declare that the research was conducted in the absence of any commercial or financial relationships that could be construed as a potential conflict of interest.

## References

[1] Lei C Ma S Huang M An J Liang B Dai J. Long-term survival and late toxicity associated with pelvic intensity modulated radiation therapy (IMRT) for cervical cancer involving CT-based positive lymph nodes. Front Oncol. (2019) 9:520. 10.3389/fonc.2019.00520 31275853 PMC6593063

[2] Chen HH Yu HI Cho WC Tarn WY. DDX3 modulates cell adhesion and motility and cancer cell metastasis via Rac1-mediated signaling pathway. Oncogene. (2015) 34:2790–800. 10.1038/onc.2014.190 25043297

[3] Kanai Y Oda T Tsuda H Ochiai A Hirohashi S. Point mutation of the E-cadherin gene in invasive lobular carcinoma of the breast. Jpn J Cancer Res. (1994) 85:1035–9. 10.1111/j.1349-7006.1994.tb02902.x 7961105 PMC5919349

[4] Vessey CJ Wilding J Folarin N Hirano S Takeichi M Soutter P. Altered expression and function of E-cadherin in cervical intraepithelial neoplasia and invasive squamous cell carcinoma. J Pathol. (1995) 176:151–9. 10.1002/path.1711760208 7636625

[5] Mayer A Hockel M Schlischewsky N Schmidberger H Horn LC Vaupel P. Lacking hypoxia-mediated downregulation of E-cadherin in cancers of the uterine cervix. Br J Cancer. (2013) 108:402–8. 10.1038/bjc.2012.570 23322209 PMC3566820

[6] Muthusami S Prabakaran DS Yu JR Park WY. EGF-induced expression of Fused Toes Homolog (FTS) facilitates epithelial-mesenchymal transition and promotes cell migration in ME180 cervical cancer cells. Cancer Lett. (2014) 351:252–9. 10.1016/j.canlet.2014.06.007 24971934

[7] Bae JS Noh SJ Kim KM Park SH Hussein UK Park HS. SIRT6 is involved in the progression of ovarian carcinomas via β-catenin-mediated epithelial to mesenchymal transition. Front Oncol. (2018) 8:538. 10.3389/fonc.2018.00538 30524965 PMC6256124

[8] Lim J Thiery JP. Epithelial-mesenchymal transitions: insights from development. Development. (2012) 139:3471–86. 10.1242/dev.071209 22949611

[9] Frixen UH Behrens J Sachs M Eberle G Voss B Warda A. E-cadherin-mediated cell-cell adhesion prevents invasiveness of human carcinoma cells. J Cell Biol. (1991) 113:173–85. 10.1083/jcb.113.1.173 2007622 PMC2288921

[10] Jang D Kwon H Choi M Lee J Pak Y. Sumoylation of Flotillin-1 promotes EMT in metastatic prostate cancer by suppressing Snail degradation. Oncogene. (2019) 38:3248–60. 10.1038/s41388-018-0641-1 30631151 PMC6756018

[11] Cleton-Jansen AM Callen DF Seshadri R Goldup S McCallum B Crawford J. Loss of heterozygosity mapping at chromosome arm 16q in 712 breast tumors reveals factors that influence delineation of candidate regions. Cancer Res. (2001) 61:1171–7. 11221848

[12] Price SR De Marco Garcia NV Ranscht B Jessell TM. Regulation of motor neuron pool sorting by differential expression of type II cadherins. Cell. (2002) 109:205–16. 10.1016/s0092-8674(02)00695-5 12007407

[13] Wiech T Nikolopoulos E Weis R Langer R Bartholome K Timmer J. Genome-wide analysis of genetic alterations in Barrett's adenocarcinoma using single nucleotide polymorphism arrays. Lab Invest. (2009) 89:385–97. 10.1038/labinvest.2008.67 18663352

[14] Zhou D Yang L Zheng L Ge W Li D Zhang Y. Exome capture sequencing of adenoma reveals genetic alterations in multiple cellular pathways at the early stage of colorectal tumorigenesis. PLoS ONE. (2013) 8:e53310. 10.1371/journal.pone.0053310 23301059 PMC3534699

[15] Muller E Brault B Holmes A Legros A Jeannot E Campitelli M. Genetic profiles of cervical tumors by high-throughput sequencing for personalized medical care. Cancer Med. (2015) 4:1484–93. 10.1002/cam4.492 26155992 PMC4618619

[16] Sjoblom T Jones S Wood LD Parsons DW Lin J Barber TD. The consensus coding sequences of human breast and colorectal cancers. Science. (2006) 314:268–74. 10.1126/science.1133427 16959974

[17] Wang L Wei D Huang S Peng Z Le X Wu TT. Transcription factor Sp1 expression is a significant predictor of survival in human gastric cancer. Clin Cancer Res. (2003) 9:6371–80. 14695137

[18] Yang P Su C Luo X Zeng H Zhao L Wei L. Dietary oleic acid-induced CD36 promotes cervical cancer cell growth and metastasis via up-regulation Src/ERK pathway. Cancer Lett. (2018) 438:76–85. 10.1016/j.canlet.2018.09.006 30213558

[19] Bharadwaj M Strohmeyer N Colo GP Helenius J Beerenwinkel N. αV-class integrins exert dual roles on α5β1 integrins to strengthen adhesion to fibronectin. Nat Commun. (2017) 8:14348. 10.1038/ncomms14348 28128308 PMC5290147

[20] Chaudhury A Hussey GS Ray PS Jin G Fox PL Howe PH. TGF-β-mediated phosphorylation of hnRNP E1 induces EMT via transcript-selective translational induction of Dab2 and ILEI. Nat Cell Biol. (2010) 12:286–93. 10.1038/ncb2029 20154680 PMC2830561

[21] Xu J Lamouille S Derynck R. TGF-β-induced epithelial to mesenchymal transition. Cell Res. (2009) 19:156–72. 10.1038/cr.2009.5 19153598 PMC4720263

[22] Foty RA Steinberg MS. Measurement of tumor cell cohesion and suppression of invasion by E- or P-cadherin. Cancer Res. (1997) 57:5033–6. 9371498

[23] Mareel M Bracke M Van Roy F. Invasion promoter versus invasion suppressor molecules: the paradigm of E-cadherin. Mol Biol Rep. (1994) 19:45–67. 8170467 10.1007/BF00987321

[24] Kools P Van Imschoot G van Roy F. Characterization of three novel human cadherin genes (CDH7, CDH19, and CDH20) clustered on chromosome 18q22-q23 and with high homology to chicken cadherin-7. Genomics. (2000) 68:283–95. 10.1006/geno.2000.6305 10995570

[25] Benham-Pyle BW Pruitt BL Nelson WJ. Cell adhesion. Mechanical strain induces E-cadherin-dependent Yap1 and beta-catenin activation to drive cell cycle entry. Science. (2015) 348:1024–7. 10.1126/science.aaa4559 26023140 PMC4572847

[26] Kalluri R Weinberg RA. The basics of epithelial-mesenchymal transition. J Clin Invest. (2009) 119:1420–8. 10.1172/jci39104 19487818 PMC2689101

[27] Gugnoni M Sancisi V Gandolfi G Manzotti G Ragazzi M Giordano D. Cadherin-6 promotes EMT and cancer metastasis by restraining autophagy. Oncogene. (2017) 36:667–77. 10.1038/onc.2016.237 27375021

[28] Brabletz T Kalluri R Nieto MA Weinberg RA. EMT in cancer. Nat Rev Cancer. (2018) 18:128–34. 10.1038/nrc.2017.118 29326430

[29] Yang G Liang Y Zheng T Song R Wang J Shi H. FCN2 inhibits epithelial-mesenchymal transition-induced metastasis of hepatocellular carcinoma via TGF-beta/Smad signaling. Cancer Lett. (2016) 378:80–6. 10.1016/j.canlet.2016.05.007 27177473

[30] Bocci F Jolly MK Tripathi SC Aguilar M Hanash SM Levine H. Numb prevents a complete epithelial-mesenchymal transition by modulating Notch signalling. J R Soc Interface. (2017) 14:512. 10.1098/rsif.2017.0512 29187638 PMC5721160

[31] Zhao G Wojciechowski MC Jee S Boros J McAvoy JW Lovicu FJ. Negative regulation of TGFβ-induced lens epithelial to mesenchymal transition (EMT) by RTK antagonists. Exp Eye Res. (2015) 132:9–16. 10.1016/j.exer.2015.01.001 25576668

[32] Hutchins EJ Bronner ME. Draxin acts as a molecular rheostat of canonical Wnt signaling to control cranial neural crest EMT. J Cell Biol. (2018) 217:3683–97. 10.1083/jcb.201709149 30026247 PMC6168252

[33] Shirakihara T Saitoh M Miyazono K. Differential regulation of epithelial and mesenchymal markers by deltaEF1 proteins in epithelial mesenchymal transition induced by TGF-beta. Mol Biol Cell. (2007) 18:3533–44. 10.1091/mbc.e07-03-0249 17615296 PMC1951739

[34] Schulte J Weidig M Balzer P Richter P Franz M Junker K. Expression of the E-cadherin repressors Snail, Slug and Zeb1 in urothelial carcinoma of the urinary bladder: relation to stromal fibroblast activation and invasive behaviour of carcinoma cells. Histochem Cell Biol. (2012) 138:847–60. 10.1007/s00418-012-0998-0 22820858

[35] Stewart CJ McCluggage WG. Epithelial-mesenchymal transition in carcinomas of the female genital tract. Histopathology. (2013) 62:31–43. 10.1111/his.12057 23240668

[36] Bienz M. beta-Catenin: a pivot between cell adhesion and Wnt signalling. Curr Biol. (2005) 15:R64–7. 10.1016/j.cub.2004.12.058 15668160

[37] Grzesiak M Mitan A Janik ME Knapczyk-Stwora K Slomczynska M. Flutamide alters beta-catenin expression and distribution, and its interactions with E-cadherin in the porcine corpus luteum of mid- and late pregnancy. Histol Histopathol. (2015) 30:1341–52. 10.14670/hh-11-630 25976454

