# Correction: The interaction of CDH20 With β-catenin inhibits cervical cancer cell migration and invasion via TGF-β/Smad/SNAIL mediated EMT

**DOI:** 10.3389/fonc.2026.1965794

**Published:** 2026-09-25

**Authors:** Chao Li, Hongfeng Ao, Guofang Chen, Fang Wang, Fang Li

**Affiliations:** 1 Clinical and Translational Research Center, Shanghai First Maternity and Infant Hospital, Tongji University School of Medicine, Shanghai, China; 2 Department of Pathology, Shanghai Fengxian District Central Hospital, Shanghai Jiao Tong University Affiliated Sixth People's Hospital South Campus, Shanghai, China; 3 Department of Gynecology, Shanghai East Hospital, Tongji University School of Medicine, Shanghai, China

**Keywords:** cervical cancer, cadherin 20, β-catenin, cancer metastasis, epithelial-to-mesenchymal transition, pSmad2/3

There was a mistake in **Figure 3** as published. Incorrect vertical axis scales were provided for “panels E and F” of **Figure 3**. The corrected **Figure 3** appears below.

**Figure 3:**
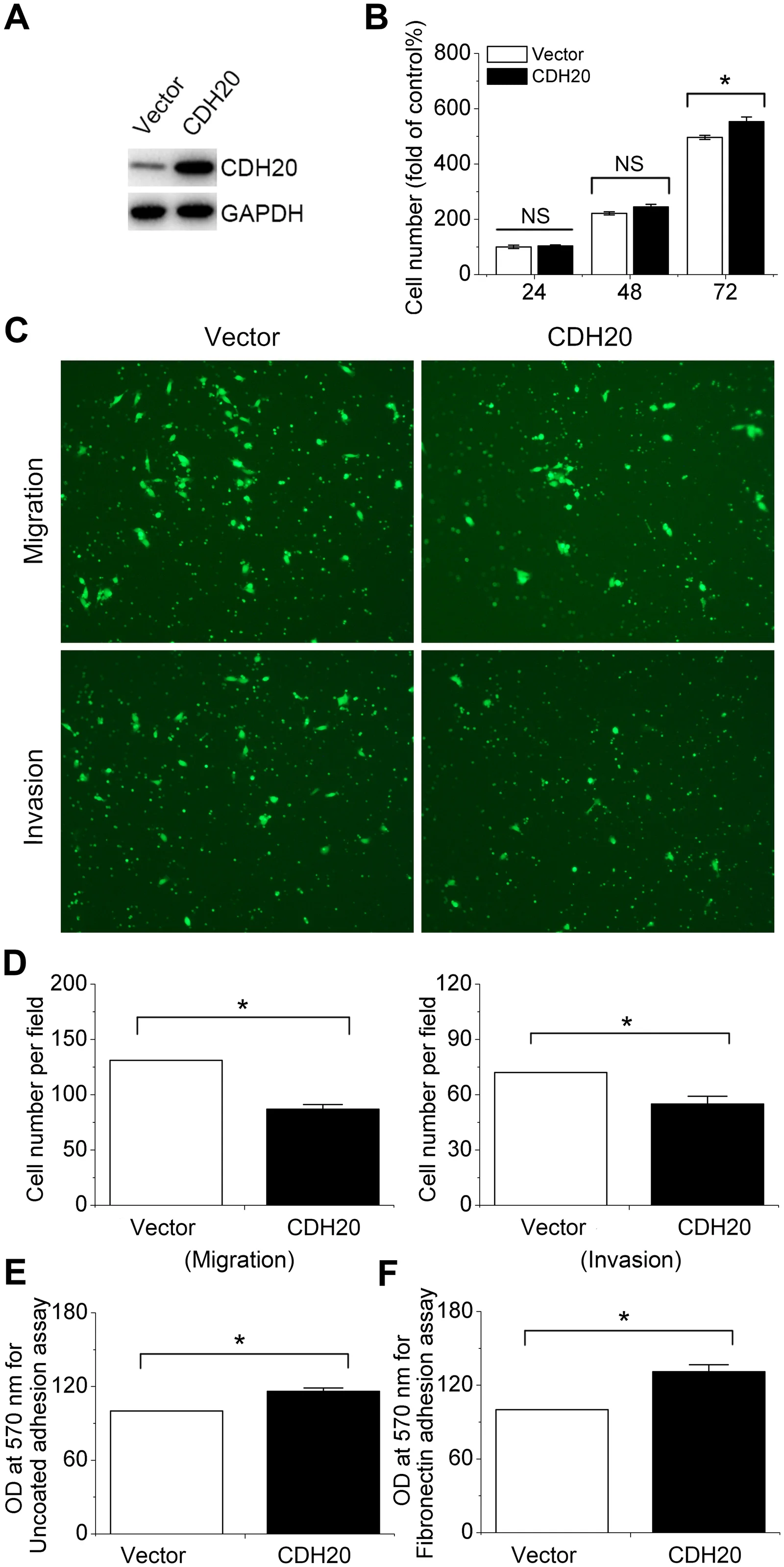
Upregulated CDH20 enhances cervical cell adhesion but reduces cell migration and invasion. Caski cell lines stably overexpressing CDH20 or empty vector were established by lentivirus infection. **(A)** The lysates were blotted with the indicated antibodies. **(B)** Proliferation of the Caski stable cell lines was detected by MTS assay. The cell proliferation index of the vector group at 24 h was defined as 100%. **(C)** Migration and invasion of Caski stable cell lines were determined by transwell assay. Representative images are shown. **(D)** Changes in cell migration and invasion were quantified. **(E)** Cell adhesion to uncoated plates was measured by cell adhesion assay using the Caski stable cell lines. **(F)** Cell adhesion to fibronectin-coated plates was measured by cell adhesion assay using the Caski stable cell lines. All statistical data were analyzed by one-way analysis of variance (ANOVA). All values represent the means ± SEM. *P < 0.05 compared with the control. NS, non-significant.

There was a mistake in **Figure 4** as published. Incorrect immunofluorescence images were provided for the first row of “panel D” in **Figure 4**. The corrected **Figure 4** appears below.

**Figure 4:**
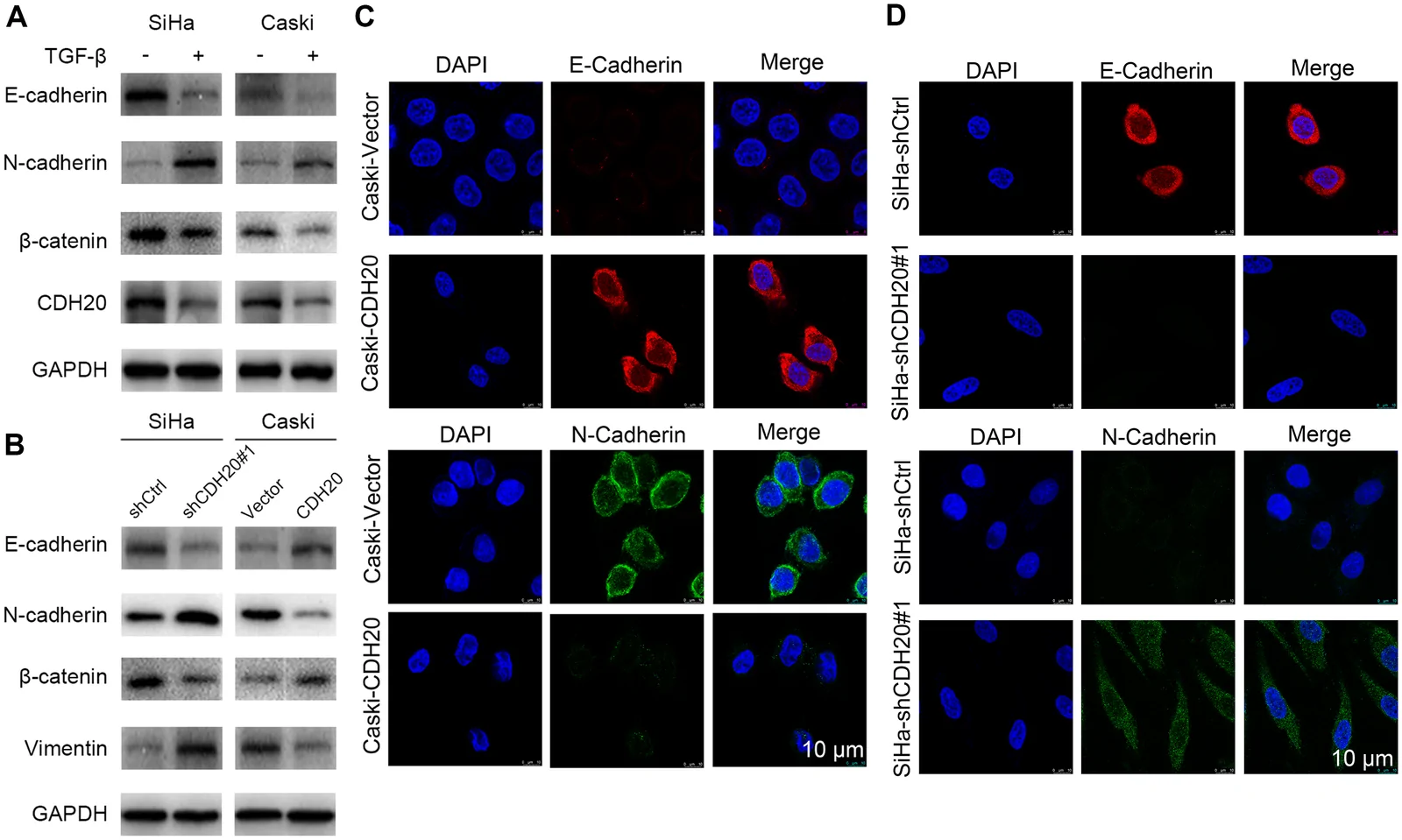
Different expression patterns of CDH20 differently affect the EMT process of cervical cancer cells. **(A)** CDH20 was downregulated in artificial EMT models induced by TGF-β in both SiHa and Caski cells. Upregulation of N-cadherin or downregulation of E-cadherin and β-catenin expression was verified in EMT models. **(B)** Western blot analysis of EMT markers (E-cadherin, N-cadherin, β-catenin, and vimentin) in the CDH20 knockdown SiHa cells or the CDH20 overexpression Caski cells compared with those in the control groups. GAPDH was used to normalize protein expression. One representative of three different experiments, for each of the analyses performed, is shown. **(C)** Immunofluorescence showed that E-cadherin and N-cadherin expression levels were increased or decreased, respectively, in CDH20-overexpressing Caski cells via laser confocal microscopy. **(D)** Immunofluorescence showed that E-cadherin and N-cadherin expression levels were decreased or increased, respectively, in CDH20-knockdown SiHa cells via laser confocal microscopy. Representative images of E-cadherin and β-catenin N-cadherin localization are shown.

The original version of this article has been updated.

