# Supplementary material for: The Interaction of CDH20 With β-Catenin Inhibits Cervical Cancer Cell Migration and Invasion via TGF-β/Smad/SNAIL Mediated EMT
Source: Front Oncol. 2020 Jan 9;9:1481. doi: 10.3389/fonc.2019.01481 (PMC6962355; doi:10.3389/fonc.2019.01481)
Supplement: Supplementary file 1 [file Table_1.DOCX]

Supplementary Material

# Supplementary Table 1 PCR primers used in this study.

| Gene | Primer name | Sequence (5' to 3') |  |
| --- | --- | --- | --- |
| CDH20 | 20-F | GGGACCGACCCTTTGTATGTC | |
|  | 20-R | CGATGGTAAACACGATGCCAG | |
| β-catenin | CTNNB-F | AAAGCGGCTGTTAGTCACTGG | |
|  | CTNNB-R | CGAGTCATTGCATACTGTCCAT | |
| GAPDH | GAPDH-F | CTGCACCACCAACTGCTTAG | |
|  | GAPDH-R | TTCTGGGTGGCAGTGATG | |
